# Retrospective Examination of Infants With Congenital Neural Tube Defect

**DOI:** 10.7759/cureus.48886

**Published:** 2023-11-16

**Authors:** İsmail Hakki Özcan, Murat Yücel, Hasan Kahveci

**Affiliations:** 1 Department of Pediatrics, Beylikdüzü State Hospital, İstanbul, TUR; 2 Department of Neurosurgery, Yalova University Medical School, Yalova, TUR; 3 Department of Pediatrics, Erzurum City Hospital, University of Health Sciences, Erzurum, TUR

**Keywords:** neural tube, infant, folic acid, hydrocephalus, neural tube defects

## Abstract

Introduction: In the present study, neonates presenting with neural tube defects (NTDs) and undergoing observation within the confines of the neonatal intensive care unit (NICU) were subjected to a comprehensive assessment encompassing concurrent morbidities, clinical manifestations, laboratory parameters, and instituted interventions.

Materials and methods: A retrospective examination was conducted on the medical records of 135 neonates diagnosed with congenital NTD within the temporal span from 2008 to 2018. The study cohort was drawn from the NICU of the Health Sciences University Erzurum Regional and Research Hospital Health Practice and Research Center.

Discussion: The current investigation encompasses a retrospective analysis of 135 patients diagnosed with NTD who received treatment at the NICU between the years 2008 and 2018. Among these, 74 individuals (54.2%) were male, while 61 (45.8%) were female. Maternal ages ranged from 17 to 46 years, with variations in the number of pregnancies, ranging from 1 to 10. Notably, 71 cases (52.6%) were delivered through normal spontaneous delivery, whereas 64 cases (47.4%) underwent cesarean section. The familial context revealed that five patients (3.6%) had siblings with a history of NTD, while no instances were noted where mothers had received antenatal folic acid support. Birth weights of the neonates ranged from 1425 to 4500 grams. Consanguinity was identified in the parental relationships of 17 cases (12.6%). The average diameter of the neural tube sac was determined to be 4.83 ± 1.94 cm (1-12 cm). Predominantly, the lumbosacral region emerged as the most common site of NTD, with meningomyelocele being the prevailing NTD type. Hydrocephalus coexisted in 67 cases, and notably, 44 instances exhibited the development of hydrocephalus post-sac operation. Eight patients were deemed inoperable, and the initial surgery transpired at an average age of 4.3 ± 2.6 (0-17) days. Flap closure constituted 32 of the surgical interventions, while primary closure was implemented in 95 cases. Neurogenic bladder antedated the operation in 14 patients, and 12 individuals developed neurogenic bladder postoperatively. Ventriculoperitoneal shunt placement was warranted in 47 patients. The average duration of hospitalization was 22.5 ± 14.4 days. Regrettably, three patients died due to complications and infections during their hospital stay.

Result: NTD represents a significant cohort of pathologies necessitating a comprehensive and interdisciplinary management strategy. These anomalies are characterized by elevated morbidity and mortality rates, not only exerting substantial financial strains on societal, familial, and state healthcare resources but also inflicting profound emotional distress upon affected families. Crucially, periconceptional strategies emphasizing balanced nutrition coupled with targeted multivitamin and mineral supplementation, particularly the inclusion of folic acid, assume paramount importance in the prophylaxis of this debilitating condition.

## Introduction

Congenital anomalies stand as the predominant cause of mortality within the first year of infancy. Among these anomalies, neural tube defects (NTDs) exhibit an incidence of approximately one per 1000 births among white Americans, positioning them as the prevailing category of congenital anomalies subsequent to congenital heart defects. Principal subtypes within the domain of NTDs encompass spina bifida and anencephaly. The etiological underpinnings of NTDs manifest as intricate, involving a nuanced interplay of genetic and environmental factors. This complexity necessitates a comprehensive understanding, encompassing both genetic predispositions and environmental influences in delineating the pathogenesis of NTDs [1]. Anencephaly ensues from the non-coalescence of the cranial neural tube, resulting in a congenital anomaly marked by incomplete development of the brain and cranial vault. In contrast, meningomyelocele, colloquially referred to as spina bifida, arises due to a failure in neural tube fusion within the spinal region. This condition is characterized by the incomplete closure of the vertebral column, leading to the exposure of neural elements and associated neurological complications [2].

Based on epidemiological data spanning the years 2004 to 2006, as documented by the Centers for Disease Control and Prevention (CDC), the incidence rates for congenital anomalies are delineated as follows: spina bifida manifests at a frequency of one in 2858, anencephaly at one in 4859, and encephalocele at a rate of one in 12235 [3]. In the year 1975, Brock et al. disclosed a correlation between elevated alpha-fetoprotein levels and disorders of the central nervous system [4]. NTDs entail a spectrum of complications encompassing hydrocephalus, central nervous system infections, kyphoscoliosis, neurogenic bladder, fecal incontinence, obesity, shunt occlusion or dysfunction, nonverbal learning and executive function difficulties, psychosocial morbidity, urinary infections, vesicoureteral reflux, hydronephrosis, syringomyelia, attention-deficit hyperactivity disorder, cardiovascular diseases, lymphedema, and other complexities. Surgical interventions have been observed to enhance survival rates when conducted at an appropriate juncture [5]. The prevalence of NTDs has exhibited a decline concomitant with the implementation of periconceptional folic acid supplementation [6]. The American Academy of Pediatrics aligns with the US Public Health Service (USPHS) recommendation, advocating the daily consumption of 400 micrograms of folic acid by women of childbearing potential to mitigate the risk of NTDs. Research indicates that periconceptional folic acid supplementation yields a preventive efficacy exceeding 50% in the occurrence of NTDs, encompassing conditions such as spina bifida and anencephaly. Moreover, for individuals with a history of a prior pregnancy affected by an NTD, the CDC advises the initiation of folic acid intake for at least one month preceding conception, which has to be continued daily for three months [7]. Women with a history of pregnancies affected by NTDs are advised by the CDC to augment their folic acid intake to 4,000 micrograms per day. Commencing at least one month before conception, this heightened supplementation is recommended to persist throughout the initial trimester [7].

The management of NTDs necessitates a protracted and comprehensive treatment regimen, mandating a multidisciplinary approach. The objective of this study is to scrutinize the clinical outcomes of patients with NTDs within our cohort, juxtaposing these findings with existing literature for comprehensive analysis.

## Materials and methods

In this study, a retrospective analysis was conducted on the medical records of newborns admitted to the Newborn Clinic of the University of Health Sciences Erzurum Regional and Research Hospital (SUAM) with a diagnosis within the congenital neural tube defect disease spectrum. The study cohort comprised 135 patients diagnosed with various manifestations, including meningocele, myelocele, meningomyelocele, encephalocele, encephalomyelocele, spina bifida occulta, anencephaly, and hydrocephalus, frequently co-occurring with NTDs. The retrospective investigation spanned the years 2008 to 2018.

Data collection methodology involved a comprehensive assessment of complaints, medical history, cardiovascular profile, familial medical history, prenatal medical history, medication usage, pregnancy-related diagnoses, physical examination findings, comorbidities, and consultations from relevant departments. Imaging findings were scrutinized to evaluate concurrent hydrocephalus and anomalies in all cases. During clinical follow-up, parameters such as examination findings, head circumference, fontanel tension, repeat imaging, and consultations with various departments were systematically examined.

For patients subjected to ventriculoperitoneal shunt placement, daily evaluations encompassed head circumference, fontanel bulge, hydrocephalus-related indicators, and shunt assessments. In cases exhibiting symptoms of respiratory distress, appropriate respiratory support strategies, including oxygen administration, continuous positive airway pressure (CPAP), and mechanical ventilation, were implemented based on symptomatology and blood gas analyses.

Prophylactic antibiotic administration was undertaken in instances of suspected sac infection, shunt infection, sepsis, central nervous system infection, pneumonia, and other infectious scenarios. Antiepileptic medications were administered for prophylactic and therapeutic purposes against convulsions, with preventive treatments addressing electrolyte imbalances. Breastfeeding was encouraged for those capable, while patients with sucking and swallowing difficulties received nutrition through suitable formula via a feeding tube and parenteral routes when necessary.

Diagnostic assessments included urinary ultrasonography for suspected renal anomalies, voiding cystoureterogram for findings indicative of vesicoureteral reflux, and urodynamics for features suggestive of neurogenic bladder dysfunction. Modes of birth, gender, gestational age, birth weight, birth length, and birth head circumference were meticulously documented. Maternal characteristics, including age, number of pregnancies, live births, abortions, history of neural tube defect-affected births, drug usage during pregnancy, consanguinity between parents, maternal folic acid supplementation, maternal medical history, history of stillbirth, neural tube defect sac status and diameter, and location of the neural tube defect, were comprehensively evaluated.

Additional analyses involved the distribution of NTDs according to location and type, the occurrence of hydrocephalus postoperatively, hip dislocation, presence of concomitant somatic and cardiac anomalies, biochemical parameters (AST, ALT, sodium, potassium, BUN, creatinine), hematological parameters (white blood cell count, hemoglobin, hematocrit, platelet count), thyroid function tests (TSH, free T4), initiation of thyroid replacement therapy, inoperable cases, and patient discharge status. Further investigations encompassed details regarding the first surgical procedure, specifically whether it involved primary or flap closure, the presence of preoperative neurogenic bladder, postoperative development of neurogenic bladder, ventriculoperitoneal shunt placement, antibiotic content in the ventriculoperitoneal shunt, duration of hospitalization, instances of extraventricular drainage set placement, treatment duration, and in-hospital mortality.

The acquired research data underwent classification using the Statistical Package for the Social Sciences (SPSS) for Windows statistics software, version 16.9 (IBM Corp., Armonk, NY). Subsequently, a comprehensive frequency table delineating the demographic attributes of the study participants was meticulously generated. The statistical analysis of the research data involved the application of measures such as mean, standard deviation, and frequency distribution tests to analyze specific scales.

## Results

Our study encompasses 135 cases, with 74 (54.8%) identified as male and 61 (45.2%) as female. Examination of the demographic characteristics of the mothers reveals a range of birth weights for the infants from 1425 to 4500 g. Maternal ages spanned between 17 and 46 years, with the number of pregnancies ranging from one to 10. The birth length of the infants ranged from 42 to 51 cm, and the birth head circumference varied from 28 to 45 cm. Notably, mothers were identified as being at risk of miscarriage, with the recorded risk occurrences falling within the range of 0 to 6 (Table 1).

**Table 1:** Descriptive statistics of patient's mothers' birth weeks, birth weights, maternal ages, number of pregnancies, birth lengths, birth head circumferences, and number of maternal miscarriages

	N	Minimum	Maximum	Average	Standard deviation (±)
Gestational age	135	19	42	38,41	2,67
Number of abortions	135	0	6	0,3704	0,91
Mother's age	135	17	46	27,07	5,97
Number of pregnancies	135	1	10	3,13	2,27
Birth weight (g)	135	1425	4500	3049,84	475,54
Birth height	135	42	51	47,67	1,83
Birth head circumference	135	28	45	36,29	2,27

Analysis of the birth types among the mothers indicated that 71 (52.5%) opted for normal spontaneous vaginal birth (NSVD), while 64 (47.5%) underwent cesarean section (C/S). Further examination of parental relationships revealed that 17 (12.7%) were consanguineous, while 117 (87.3%) were not.

Regarding medication usage among mothers, the majority, 127 (94.8%), did not utilize any medication. Among those who did, five (3.7%) used iron supplements, one (0.7%) used multivitamins, and another one (0.7%) used antiepileptic medication.

Infant weights at birth were categorized as follows: two (1.5%) weighed less than 1500 grams, 12 (8%) fell within the 1500-2500 grams range, 117 (88%) were within the 2500-4000 grams range, and two (1.5%) exceeded 4000 grams (Table 1).

Maternal age distribution revealed that 11 (8.2%) were under the age of 20, 42 (31.3%) were between 20 and 24 years, 39 (29.1%) were between 25 and 29 years, 26 (19.4%) were between 30 and 35 years, 15 (11.2%) were between 36 and 45 years, and one (0.7%) was older than 45 years. In terms of health status, the majority of mothers, 133 (98.4%), reported no preexisting conditions, while one (0.8%) had diabetes and another one (0.8%) had epilepsy.

The integrity of the NTD sac was observed to be intact in 111 (82%) cases and defective in 24 (18%) cases. In our study cohort of 135 cases, the distribution of NTD locations revealed a diverse pattern. Specifically, two cases (1.5%) manifested in the cervical region, three cases (2.3%) in the thoracic region, and 44 cases (33.1%) exhibited lumbar involvement. Additionally, six cases (4.5%) displayed thoracolumbar involvement, while 69 cases (51.9%) presented with NTDs in the lumbosacral region. A singular case (0.8%) appeared sacrococcygeal, and five cases (3.8%) were observed in the sacral region. Further, three cases (2.3%) were identified with NTDs in the occipital region. This comprehensive distribution analysis provides insights into the varied anatomical locations of NTD occurrences within our study population. Examining the distribution of NTD types within our study cohort of 135 cases yielded diverse manifestations. Specifically, one case (0.7%) presented with meningocele, two cases (1.5%) with myelocele, and the majority, 118 cases (87.4%), exhibited the meningomyelocele phenotype. Additionally, five cases (3.7%) displayed encephalocele, two cases (1.5%) manifested encephalomyelocele, two cases (1.5%) presented spina bifida occulta, and another two cases (1.5%) demonstrated co-occurrence of spina bifida and dermal sinus. Moreover, three cases (2.2%) were identified as anencephaly.

Further scrutiny of the dataset unveiled that 67 cases (49.6%) were concomitant with hydrocephalus, while the remaining 68 cases (50.4%) were devoid of hydrocephalic complications. This detailed categorization delineates the nuanced landscape of NTD types and their associated hydrocephalic presentations within our study population. Analysis of the postoperative outcomes revealed that hydrocephalus ensued following the pouch operation in 44 cases (34.6%), while it did not manifest in the remaining 83 cases (65.4%). Furthermore, five cases (3.7%) exhibited hip dislocation, contrasting with the majority of 130 cases (96.3%) devoid of this complication.

In delineating somatic anomalies concomitant with NTD, a diverse array of manifestations was observed. Specifically, pes equinovarus was identified in seven cases (28%), Arnold Chiari malformation in two cases (8%), hip dislocation in one case (4%), and hypospadias in one case (4%). Additionally, left knee deformity was noted in two cases (8%), hydronephrosis in two cases (8%), atresia of the external auditory canal in one case (4%), and anal atresia and scrotal hernia in two cases (8%). Four cases (16%) presented with congenital rickets, while two cases (8%) exhibited the coexistence of pes equinovarus and hip dislocation. Notably, one case (4%) featured an intrauterine femur fracture. This nuanced exploration underscores the spectrum of somatic anomalies that may accompany NTD, providing comprehensive insights into the clinical landscape. Upon scrutinizing the distribution of transthoracic echocardiography findings among patients with NTDs, it was discerned that the echocardiographic assessments of seven patients revealed the presence of hemodynamically significant pathologies during the neonatal period.

Of the total cases investigated, eight instances (5.9%) were deemed inoperable, while the majority, 127 cases (94.1%), underwent surgical intervention. The timing of the initial surgeries varied, with two cases (2%) occurring on the first day of life, 27 cases (27%) on the third day, 34 cases (34%) on the fourth day, 13 cases (13%) on the fifth day, five cases (5%) on the sixth day, one case (1%) on the seventh day, five cases (5%) on the eighth day, two cases (2%) on the ninth day, two cases (2%) on the 10th day, three cases (3%) on the 11th day, one case (1%) on the 12th day, and one case (1%) on the 13th day. Primary closure surgery was undertaken in 94 cases (69%), while 33 cases (31%) underwent flap closure surgery. A preoperative assessment revealed that 14 cases (10.3%) presented with a neurogenic bladder, while the majority, 113 cases (89.1%), did not exhibit this condition. Postoperatively, neurogenic bladder developed in 12 cases (9.4%), while 115 cases (90.6%) remained unaffected. Ventriculoperitoneal shunt placement was performed in 47 cases (37%), while 80 cases (63%) did not undergo this procedure. Analysis of the neurological status of the lower extremity before surgery indicated that 42 cases (32.8%) presented with paraplegia, whereas 85 cases (67.2%) did not exhibit this condition. Postoperatively, 80 cases (63%) experienced no loss of strength or paraplegia in the lower extremity, 25 cases (19.6%) exhibited partial loss of strength, and 22 cases (17.3%) displayed complete paralysis.

Hypothyroidism was diagnosed in 8.3% of patients, prompting the initiation of thyroid replacement therapy. There were no substantial deviations in hemogram and biochemistry parameters when compared to the normal population (Table 2).

**Table 2:** Laboratory findings of the cases

	N	Minimum	Maximum	Average	Standard deviation (±)
Aspartate transaminase (AST), IU/L	135	12	88	33,94	12,81
Alanine transaminase (ALT), IU/L	135	7	67	26,30	10,60
Sodium, mmol/L	135	133	148	140,30	3,03
Potassium, mmol/L	135	3,7	6,41	4,66	0,53
Blood urea nitrogen, mg/dL	135	4,4	54,4	16,88	6,51
Creatinine, mg/dL	135	0,1	2,38	0,49	0,22
White blood cell value (piece/m^3^)	135	4660	36200	14515,15	3241,03
Hemoglobin, gr/dL	135	3,95	23	14,59	2,32
Hematocrit, %	135	23,4	67	45,13	5,80
Thrombocyte (piece/mm^3^)	135	156000	724000	363267,42	111157,20
Free tiroxine (ft^4^), ng/dl	79	0,4	2,24	1,25	0,35
Thyroid-stimulating hormone (TSH), mU/L	79	0,18	114	13,16	22,90
Free thyroxine (fT4) (second test), ng/dl	16	0,75	3,45	1,6	0,70
Thyroid-stimulating hormone (second test), mU/L	16	0,68	36,94	5,96	8,57

## Discussion

Despite the contemporary decline in the incidence of NTDs, they persist as one of the most prevalent congenital anomalies affecting newborns. These anomalies contribute to a spectrum of challenges, including disabilities, central nervous system infections, skeletal disorders, kidney anomalies, and nutritional intolerances, all of which exert a discernible impact on the overall quality of life. Several factors have been identified as potential contributors to the prevalence of NTDs, encompassing parental history of NTDs, prior occurrence of such defects in a sibling, folic acid deficiency, genetic predispositions such as MTHFR gene defects and homocysteinemia, low socioeconomic status, and maternal use of certain medications, including antiepileptics like carbamazepine and valproate as well as drugs such as isotretinoin, aminopterin, thalidomide, and methotrexate. Additionally, poorly controlled diabetes mellitus, gestational hyperthermia, fever during pregnancy, and low socioeconomic status have been identified as potential contributors to the frequency of NTDs.

Gender-based analyses in NTD studies have revealed a subtle predominance of female cases. For instance, in a study conducted by Deak et al., the male-to-female ratio was reported as 0.82 [8]. In contrast, our study observed a slightly elevated male-to-female ratio of 1.18. In the investigation conducted by Mutlu et al., the predominant NTD observed was meningomyelocele, constituting 84.8% of the cases [9]. In our study, meningomyelocele also emerged as the most prevalent NTD, accounting for 87.4% of occurrences. Following meningomyelocele, encephalocele was identified in 3.7% of cases and anencephaly in 2%.

Notably, none of the patients in our study were utilizing folic acid supplements. This observation aligns with broader trends indicating suboptimal adherence to the US Public Health Service (USPHS) recommended levels of folic acid consumption among women. As less than one-third of women currently meet the recommended folic acid intake, the academy emphasizes the urgency of implementing effective campaigns to address this gap and prevent NTDs [7].

A related study conducted in Turkey by Oner et al. evaluated the biochemical folic acid levels in adolescent girls in Edirne. The findings revealed that 37.6% of the adolescent girls exhibited sufficient folic acid levels (≥6 ng/ml), while 46% had borderline levels (3-5.9 ng/ml), and 16.3% demonstrated insufficiency. This underscores the need for targeted interventions to enhance folic acid levels, particularly among populations at risk of NTDs [10]. In a study conducted by Aydınlı et al., a cohort of 190 individuals with a history of delivering children with NTD were administered 5 mg of folic acid and multivitamin preparations, commencing no later than two months before pregnancy and continuing for a minimum of the first two months of gestation. As a control group, 173 individuals with a history of delivering a child with NTD, yet lacking a history of preconceptional folic acid and multivitamin use, were identified. Notably, among the 190 cases subjected to preconceptional folic acid and multivitamin treatment, there were no observed instances of NTD recurrence. In contrast, the control group exhibited a recurrence rate of 4% (seven cases). Conclusively, the study findings led the authors to advocate for women with a history of NTD-affected pregnancies to initiate a regimen of at least 5 mg of folic acid a minimum of eight weeks preceding their subsequent conception, persisting until the eighth week of pregnancy at the earliest [11].

As outlined in the Risky Pregnancies Management Guide published by the Department of Women's and Reproductive Health of the Ministry of Health, Public Health Institution of Turkey, a recommended strategy to minimize potential malformations in infants involves the initiation of 400 mcg of folic acid support three months before conception, persisting until the conclusion of the 10th week of pregnancy. Notably, international studies have consistently demonstrated a significant reduction, by 50% or more, in the incidence of NTDs through periconceptional folic acid supplementation [7]. In a study conducted by Baykan et al. in 2009, the knowledge levels of 1083 women regarding folic acid and NTD were assessed. The findings revealed that 46.3% of women possessed information about folic acid, albeit fewer were informed about the specific association between folic acid and NTD. Primary sources of information were identified as healthcare personnel and the media. Strikingly, only 3.5% of non-pregnant women reported daily folic acid intake. The study further observed that knowledge levels tended to diminish with advanced age and lower educational attainment. These insights underscore the imperative of targeted educational initiatives to enhance awareness and adherence to periconceptional folic acid supplementation among women of reproductive age [12]. Within our study cohort, shunt infection manifested in 3.2% of patients, a notably lower incidence compared to the findings reported by Lee et al., where shunt infection prevalence reached 10.5% [13]. Noteworthy is the proposition that the application of topical vancomycin may serve to mitigate the risk of shunt infection [14].

In a study by Demir et al., prophylactic measures were implemented in the form of topical rifampicin (10 mg/kg/day) and cefotaxime (50 mg/kg/day, two doses, intravenous) administered to 30 newborns diagnosed with overt NTDs. In contrast, the control group, also diagnosed with overt NTD, received solely cefotaxime (50 mg/kg/day, two doses, intravenous). The findings indicated that the prophylactic topical application of rifampin on the preoperative mesh in newborns with open NTD led to a reduction in the rates of postoperative surgical site infection (SSI) and meningitis/ventriculoperitoneal shunt infection [15].

Additionally, our study observed an average maternal age of 27.1, providing contextual demographic information within the investigated population. Empirical data derived from a published study substantiate the hypothesis that maternal age, particularly those aged 40 and above, is associated with an increased risk of giving birth to a baby with NTDs. Intriguingly, this effect exhibits greater prominence in cases of spina bifida compared to anencephaly. Conversely, there is evidence indicating that mothers aged 19 years or younger face an elevated risk of delivering a child with spina bifida [16].

Within our study cohort, 52.6% of infants presenting to the clinic were delivered through normal spontaneous vaginal delivery. Existing literature suggests that infants with breech presentation and NTDs may benefit from cesarean section, although no definitive evidence supports improved outcomes in vertex presentations. In cases where the sac size exceeds 6 cm, a cesarean section may be deemed necessary to mitigate the risk of rupture. Vaginal birth is deemed suitable in all other scenarios to minimize maternal morbidity [16].

Moreover, the average weight of infants in our study ranged from 1425 to 4600 grams, with an average of 3049.9 grams. Notably, there is suggestive evidence that NTDs may contribute to intrauterine growth retardation [17]. Additionally, it has been posited that maternal weight gain both before pregnancy and during pregnancy correlates with an increased incidence of NTDs [18,19]. Cadmium-induced NTDs have been documented to elicit fetal growth restriction. This occurrence has been linked to compromised folate transport to the placenta [20]. Within a study context, malformations were identified in 8%-11% of fetuses [21]. Contrastingly, our investigation revealed accompanying somatic anomalies in 18.5%, with pes equinovarus identified in up to 5.2%. Notably, the prevalence of additional anomalies within our study cohort appears to surpass the reported figures in existing literature. While examining the distribution of NTDs in our study, the lumbosacral region emerged as the most common site at 51.1%, followed by the lumbar region at 33.3%, the sacral region at 5%, and the thoracolumbar region at 4.4%. In a study by Aygün et al., the distribution was reported as 36% lumbosacral, 30% lumbar, 18% thoracolumbar, and 6% thoracic [22]. In the investigation conducted by Eseoğlu et al., the average size of the meningomyelocele sac was documented as 4.7 cm x 5.8 cm, with a range spanning from 1 cm x 1 cm to 10 cm x 8 cm [23]. In our current study, the sac size exhibited considerable variability, ranging from 1 to 12 cm, with an average diameter of 4.8 ± 1.9 cm. Significantly, the size of the NTD holds clinical relevance in assessing the neurological prognosis of affected individuals.

Within our patient cohort, 6.5% exhibited hemodynamically significant cardiac pathology, reinforcing the established association between NTDs and an elevated risk of cardiac complications [24]. Hypothyroidism was diagnosed in 8.3% of patients, prompting the initiation of thyroid replacement therapy. This aligns with existing literature indicating an increased incidence of hypothyroidism in the presence of birth defects and NTDs [25]. Remarkably, there were no substantial deviations in hemogram and biochemistry parameters when compared to the normal population, underscoring the nuanced health profile observed in this cohort. İlhan et al. observed a shorter hospital stay in the group undergoing surgery within the first 72 hours of life compared to the group operated on after the 72nd hour of life; however, no statistically significant difference was identified between the groups [26]. Oncel et al. asserted that early surgical intervention (within five days) reduces hospital stay, antibiotic treatment duration, and complication rates, advocating for prompt corrective surgery. In our study, while the duration of the operation ranged from 0 to 17 days, the median operation time was identified as 4.25 ± 2.6 days. The average length of hospital stay was determined to be 22.4 ± 14.4 days, with babies being hospitalized on an average of 1.3 times [27]. A cohort study indicates that early myelomeningocele repair contributes to a reduction in the infection rate [20]. In our study, the duration of antibiotic use was 14.7 ± 8.1 days. Bülbül et al.'s investigation on patients with NTDs found the duration of antibiotic use to be 7.2 ± 3.2 days. Primary surgical closure was applied to 95 (73.4%) patients in our study, while 32 (26.6%) patients underwent flap closure [28]. Patterson's study reported that approximately 75% of myelomeningocele defects were closed by direct repair, with the remaining 25% requiring alternative reconstructive options [29]. Matuszczak et al.'s study identified hydrocephalus (60.6%) as the most common postoperative complication. In our study, the hydrocephalus rate was found to be 49.6% [30].

This study has limitations; conducting studies on a larger scale at a global level will contribute more to the literature.

## Conclusions

NTDs represent a significant cluster of disorders necessitating a comprehensive, interdisciplinary approach. Characterized by elevated morbidity and mortality rates, these conditions impose substantial financial burdens on individuals, families, and the state, thereby causing profound devastation within affected families. The preventive strategy against these disorders underscores the significance of periconceptional well-rounded nutrition, along with essential multivitamin and mineral supplementation, with particular emphasis on folic acid support.
